# ‘Molecular Beam Epitaxy’ on Organic Semiconductor Single Crystals: Characterization of Well-Defined Molecular Interfaces by Synchrotron Radiation X-ray Diffraction Techniques

**DOI:** 10.3390/ma15207119

**Published:** 2022-10-13

**Authors:** Yasuo Nakayama, Ryohei Tsuruta, Tomoyuki Koganezawa

**Affiliations:** 1 Department of Pure and Applied Chemistry, Tokyo University of Science, 2641 Yamazaki, Noda 278-8510, Japan; 2 Division of Colloid and Interface Science, Tokyo University of Science, Noda 278-8510, Japan; 3 Research Group for Advanced Energy Conversion, Tokyo University of Science, Noda 278-8510, Japan; 4 Faculty of Pure and Applied Sciences, University of Tsukuba, 1-1-1 Tennodai, Tsukuba 305-8577, Japan; 5 Industrial Application Division, Japan Synchrotron Radiation Research Institute (JASRI), Hyogo 679-5198, Japan

**Keywords:** organic semiconductor, p–n junction, epitaxial growth, grazing-incidence wide-angle X-ray scattering

## Abstract

Epitaxial growth, often termed “epitaxy”, is one of the most essential techniques underpinning semiconductor electronics, because crystallinities of the materials seriously dominate operation efficiencies of the electronic devices such as power gain/consumption, response speed, heat loss, and so on. In contrast to already well-established epitaxial growth methodologies for inorganic (covalent or ionic) semiconductors, studies on inter-molecular (van der Waals) epitaxy for organic semiconductors is still in the initial stage. In the present review paper, we briefly summarize recent works on the epitaxial inter-molecular junctions built on organic semiconductor single-crystal surfaces, particularly on single crystals of pentacene and rubrene. Experimental methodologies applicable for the determination of crystal structures of such organic single-crystal-based molecular junctions are also illustrated.

## 1. Introduction

Semiconductor technologies have been developed at a synchronized pace with an advance in methodologies for the fabrication of semiconductor solids toward *ideal* qualities. Molecular beam epitaxy (MBE) is an established technique to produce high-quality crystalline thin films of conventional semiconductor materials and has been widely adopted from a lab scale to industrial processes [1]. In the case of so-called organic electronics, which utilize molecular solids as semiconductors, control of the crystalline order of molecular solids is a crucial subject for the pursuit of efficiencies, reproducibilities, and stabilities of the devices. For instance, the usage of molecular single crystals rather than amorphous or polycrystalline solids has led to dramatic enhancement of the charge carrier mobility by several orders of magnitude [2].

Regarding organic optoelectronics such as organic light emitting diodes (OLEDs) and organic photovoltaics (OPVs), the performance of the devices is dominated not only by the molecular solids themselves but also by embedded interfaces between two molecular species of contrasting electronic characters, i.e., donors and acceptors, in other words, p-type and n-type organic semiconductors. In fact, the presence of donor–acceptor interfaces is essential as “p–n junctions” for practical OLED and OPV devices [3,4], and understanding and smarter engineering of the interface properties have driven the progress in the development of organic electronics [5,6,7,8,9]. Despite the particular importance of the characteristics of individual molecules for the organic semiconductors rather than continuous solid-state media, however, experimental works for the interface properties have mostly witnessed macroscopic phenomena so far, and microscopic insights in the molecular scale are still unresolved in the present stage. One central reason for this situation is that the practical interfaces are disordered systems because most organic electronic devices consist of polycrystalline or amorphous solids. Therefore, the molecular circumstances are quite inhomogeneous at the molecular levels, and essential processes driving the device performance must be obscured by the statistical ensembles of the individual molecular contacts.

Single-crystal surfaces can be used to construct well-defined model interfaces between organic semiconductors for the pursuit of molecular-scale understanding of the inter-molecular contacts. However, experimental studies to fabricate and characterize inter-molecular interfaces on molecular single-crystal surfaces were quite limited except for several pioneering works by Sassella et al. [10,11] and Yan et al. [12,13]. One reason for this situation could be ascribed to a shortage of orthodox methodologies for the characterization of the surfaces and interfaces of molecular single-crystal samples. However, a couple of successful works on precise characterizations of electronic [14] and crystallographic [15] structures performed in the late 2000s on organic semiconductor single-crystal surfaces eventually made breakthroughs in challenging this subject, stimulating several leading works by Nakayama et al. [16,17] and Miyadera et al. [18] on hetero-molecular interfaces formed on the organic semiconductor single-crystal substrates.

This article is devoted to reviewing the recent works for the formation of well-ordered epitaxial molecular junctions by an MBE-like framework on organic semiconductor single-crystal substrates, particularly on single crystals of pentacene and rubrene, and to instruct experimental methodologies and know-hows for accurate determination of the crystallographic structures of the inter-molecular junctions using “third-generation” synchrotron radiation light source (especially SPring-8). In the next section, the essences of the experimental methods for the fabrication of the molecular single-crystal substrates and surface X-ray diffraction techniques on the molecular single-crystal samples are described. Epitaxial growth manners of three molecular species of different structural characteristics on the single crystal of pentacene are reviewed in Section 3. On the other hand, on the single-crystal rubrene, three different types of epitaxy, that is, heteroepitaxy, homoepitaxy, and quasi-homoepitaxy, are illustrated in Section 4. Finally, a summary of these works and perspectives for related research fields are given in Section 5. It has to be emphasized that the authors never intended to cover a broad research field relating to epitaxial crystal growth of organic semiconductor materials but to concentrate on a very limited research topic in this paper, i.e., epitaxy of small molecules on organic semiconductor single-crystal surfaces. One can find a number of fine review articles for the related topics: e.g., construction of organic-organic heterojunctions [19], MBE of organic semiconductor molecules on inorganic single-crystal substrates [20,21], and molecular epitaxy for weakly interacted van der Waals systems [22,23,24].

## 2. Experimental Methods

### 2.1. Physical Vapor Transport

**Figure 1:**
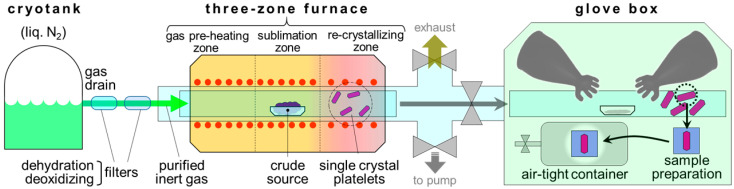
Schematic drawing of a PVT apparatus for fabrication of high-purity organic semiconductor single-crystal substrates.

Single-crystal samples of fine quality and high purity are preconditions for successful MBE. There are several methods for the fabrication of molecular single-crystal samples [25,26]. To study the well-defined molecular interfaces even by ordinary experimental methodologies, plate-shaped crystals of wide areas, thin and uniform thicknesses, and flat tops are appropriate as substrates. Physical vapor transport (PVT) is an often-used technique for yielding molecular single crystals satisfying these conditions [27]. PVT is a method to obtain single crystals by recrystallization of sublimed molecules in an inert gas stream (carrier gas) flowing in a quartz tube with a temperature gradient, which often shares the common equipment to train sublimation. The crystal growth under proper temperature settings also serves as purification of the material; impurities of lower sublimation temperatures are carried away far downstream from the recrystallization zone of the target substance, while those of high sublimation temperatures remain in the original crude source. For the inert carrier gas, nitrogen and argon are generally adopted, and hydrogen is occasionally blended to make a reducing atmosphere. In any case, the purity of the carrier gas is a crucial factor for PVT crystal growth. Therefore, the carrier gases from cylinders are supplied through dehydration and deoxidizing filters to the PVT tubes. Drain gas from cryogenic tanks is also an adequate source for high-purity carrier gas. To prevent (surface) oxidation of obtained single-crystal samples by exposure to the ambient atmosphere [28,29], it is an efficient way to connect the PVT tube to a glovebox filled with an inert gas [30,31]. Figure 1 illustrates an example of a PVT apparatus for the fabrication of high-purity organic semiconductor single-crystal samples.

### 2.2. Grazing Incidence X-ray Diffraction

Grazing incidence X-ray diffraction (GIXD), often cited as grazing incidence wide-angle X-ray scattering (GIWAXS), is a technique to analyze crystal structures of thin films. In comparison to electron diffraction techniques such as low-energy electron diffraction (LEED) and reflection high-energy electron diffraction (RHEED), GIXD is a more applicable technique for “fragile” materials to electron beam irradiation and thus has been utilized in molecular or polymer semiconductors [32,33] and recently emerging hybrid perovskite compounds [34]. In addition, whereas LEED and RHEED experiments require ultra-high vacuum conditions, GIXD can be performed in ambient conditions which are quite advantageous for in situ analyses [35]. Synchrotron radiation (SR) has rich benefits of ultra-high brightness, coherent and highly directional beam path, energy (wavelength) tunability, and so on, as a preferable X-ray source for the GIXD experiments [36].

**Figure 2:**
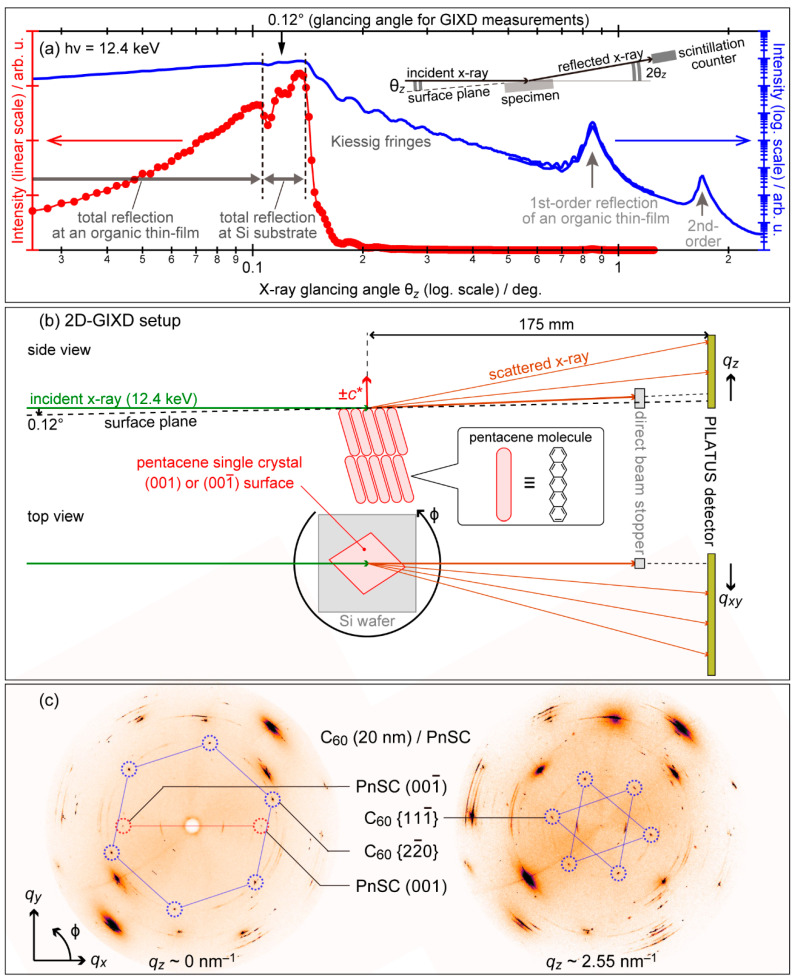
(**a**) Typical X-ray reflectivity profiles of a thin film of an organic semiconductor material spread over a Si wafer piece. (**b**) Schematic drawings of the experimental setup for the 2D-GIXD measurements. Cited from [37]. (**c**) Polar maps of the 2D-GIXD data for epitaxial C_60_ on a PnSC sample displayed in two “cuts” at constant *q_z_* positions.

In Figure 2a, X-ray reflectance at an organic molecular thin film deposited onto a Si wafer piece is plotted as a function of the X-ray glancing angle θ*_z_* with respect to the surface. A sudden drop of the reflectance at θ*_z_* = 0.14° corresponds to the total-reflection critical angle of the Si wafer for the X-ray, while a small dip at θ*_z_* = 0.105° can be attributed to that of the organic molecular film. For the sake of emphasizing the signal from the molecular thin films of typically several-tens nm-thick out of that from Si, an X-ray glancing angle that is shallower than the total-reflection critical angle of Si but is greater than that of organic thin films, for instance, 0.12°, is generally adopted for the GIXD measurements. The highly directional character of SR enables such precise control of the X-ray beam path. A typical experimental setup for the two-dimensional grazing incidence X-ray diffraction (2D-GIXD) technique is shown in Figure 2b. The scattered X-ray by a specimen is monitored using a two-dimensional detector placed perpendicular to the X-ray incident direction. The specimen is mounted on a six-axes goniometer stage which allows rotation of the azimuthal angle ϕ of the sample as well as fine alignment of the sample position and orientation with respect to the X-ray. More accurate determination of the crystal structures and evaluation of the crystallographic quality (i.e., mean crystallite size) of the samples are enabled by scanning a diffraction angle 2θ of an X-ray detector to trace a spot profile at each sample orientation. For this measurement mode, a scintillation counter of, e.g., NaI and LaBr_3_ equipped with doubled Soller slits and an X-ray attenuator is generally used as a “zero-dimensional (0D)” X-ray detector. An analyzer crystal (e.g., Ge(111) and Si(111)) is additionally inserted in between the two slits for high-resolution grazing incidence X-ray diffraction (HR-GIXD) measurements using intense and highly aligned X-ray from undulator beamlines. It should be noted that the reciprocal lattice vector perpendicular to the surface is in principle not accessible by GIXD. Instead, the crystal structures in the surface normal direction have to be measured by θ–2θ scans which are in the same manner as standard X-ray diffraction (XRD) measurements along the surface normal direction. Such out-of-plane XRD measurements are capable of using an identical machine setup to GIXD (e.g., Figure 2a inset).

For the most results reviewed in this article, the GIXD experiments were performed at BL19B2 or BL46XU of SPring-8, Japan, unless otherwise noted. The X-ray energy and glancing angle were set at 12.4 keV and 0.12° from the surface plane, respectively. A 2D X-ray detector (PILATUS300K) was located at ca. 175 mm from the ϕ rotation center of the sample for the 2D-GIXD measurements. On the single-crystalline and/or epitaxial thin-film samples, 2D-GIXD patterns were collected by rotating ϕ by 360° for the identification of the in-plane crystallographic orientation of the sample. This is a kind of tomography that (in principle) enables full mapping of the interface diffraction patterns in the three-dimensional reciprocal space for single-crystalline samples. As an example, two constant-*q_z_* “cuts” from a series of φ-dependence of the 2D-GIXD data taken on an epitaxial interface of C_60_ grown on a single crystal substrate of pentacene (details see §3-1) are presented in Figure 2c. On the other hand, for the “0D” GIXD measurements, the scintillation counter moved on a sphere with a radius of approx. 1 m centered at the sample. Distances to the first and second slits from the sample were about 480 mm and 940 mm, respectively. The angular resolution for this double-slit setup at BL19B2 was estimated to be around 0.01° [38] when the widths of the first and second slits were set at 0.2 and 0.4 mm, respectively, whereas it was broadened to be 0.04° by opening the double-slit width to 0.5 mm [39]. In contrast, the insertion of a Ge(111) analyzer crystal made the angular resolution as fine as 0.003° enabling the HR-GIXD measurements at an undulator beamline BL46XU [40].

## 3. Heteroepitaxy on Pentacene Single Crystals

### 3.1. C_60_ Fullerene/Pentacene Single Crystal

**Figure 3:**
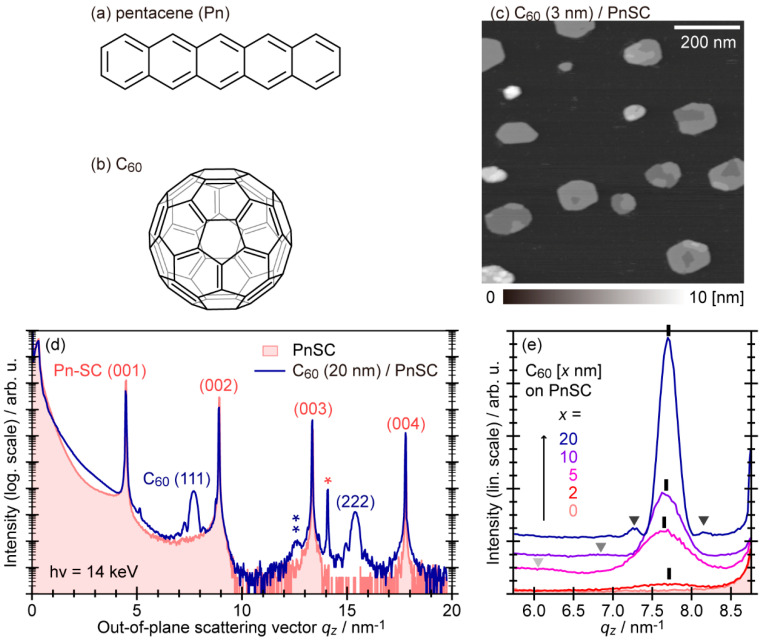
(**a**) Molecular structure of Pentacene. (**b**) Molecular structure of C_60_. (**c**) AFM topography of 3 nm-thick C_60_ deposited on a PnSC substrate. Single and double asterisk marks were ascribed to signals from misoriented crystallites. (**d**) Out-of-plane XRD profiles of a PnSC sample before (red) and after (blue) coverage of 20 nm-thick C_60_. (**e**) Evolution of the XRD profiles depending on the C_60_ thickness on PnSC. (**c**–**e**) cited from [37].

Pentacene (C_22_H_14_: Figure 3a), a typical p-type organic semiconductor material, is known to exhibit several structural phases; that is, thin film phase (*a* = 0.593 nm, *b* = 0.756 nm, *c* = 1.565 nm, α = 98.6°, β = 93.3°, γ = 89.8°) [41], bulk phase (*a* = 0.6079 nm, *b* = 0.7893 nm, *c* = 1.478 nm, α = 83.20°, β = 79.92°, γ = 94.40°) [42], and single crystal phase (*a* = 0.6266 nm, *b* = 0.7775 nm, *c* = 1.453 nm, α = 76.475°, β = 87.682°, γ = 84.684°) [43]. This material was brought into the limelight by the breakthrough of the charge carrier mobility of 1 cm^2^V^−1^s^−1^ for its vapor-deposited thin films with uniaxially oriented polycrystallites in the thin-film phase [44]. Furthermore, pentacene in the single crystal phase was reported to exhibit high charge mobility exceeding 50 cm^2^V^−1^s^−1^ at 225 K [45]. Because of such outstanding properties, pentacene has been considered a standard material for organic semiconductors. The charge carrier transport behaviors of pentacene have to be rooted in its electronic structures. Indeed, the formation of inter-molecular electronic bands was demonstrated by angle-resolved photoelectron spectroscopy (ARPES) measurements for several crystalline phases of pentacene solid-state films prepared by vacuum deposition [46,47,48,49] and also for the bulk single-crystal pentacene [50,51].

C_60_ fullerene (Figure 3b) is an n-type semiconductor material. Its crystal is consisted of a face-centered cubic (fcc) structure with a lattice constants *a* = *b* = *c* = 1.423 nm at room temperature [52]. C_60_ itself is known to exhibit considerable electron mobility as high as 10 cm^2^V^−1^s^−1^ in its single-crystalline thin films [53]. A combination of pentacene and C_60_ is known to constitute a basic organic thin-film solar cell [54], and many experimental and theoretical studies were conducted on the heterojunctions of these two molecular species [17,55,56,57,58,59,60]. However, most of the experimental studies have used polycrystalline thin films of pentacene as substrates, and thus the details of the intermolecular junction structure remain unclear due to the inhomogeneity of the sample structure itself.

Figure 3c shows an AFM topography of C_60_ deposited on a pentacene single crystal (PnSC) substrate at room temperature (RT) measured in vacuo (in situ) [37]. On the terraces of the PnSC surface, C_60_ assembled into table-like islands of very flat tops with mono-molecular steps, uniform heights, and straight rims commonly pointing to specific directions, suggesting good crystallinity. Out-of-plane XRD data exhibited clear peaks assignable to (111) and (222) reflections of C_60_ in its fcc phase as shown in Figure 3d. Moreover, each C_60_-derived peak was accompanied by so-called Laue oscillation on both sides, which was already visible for the 5 nm-thick C_60_ thin films (Figure 3e). These results also indicated that C_60_ formed (111)-oriented crystallite with a uniform out-of-plane coherent size on the PnSC surface. It is worth noting that AFM and XRD results on the samples after being taken out of the vacuum have confirmed that exposure to the ambient atmosphere and light did not induce any apparent structural change of this molecular heterojunction. All X-ray diffraction data presented hereafter were obtained from ex-situ measurements in the ambient conditions.

**Figure 4:**
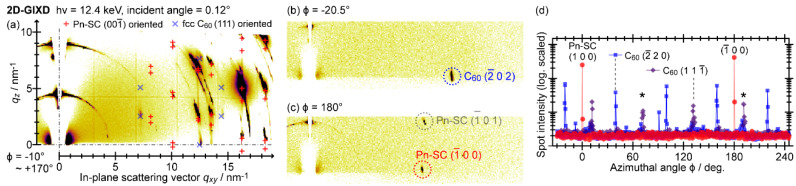
(**a**) 2D-GIXD image of a PnSC sample covered with 20 nm-thick C_60_ obtained by integration of the sample azimuthal angle over 180°. (**b**,**c**) 2D-GIXD images taken at specific sample azimuthal angles. Single marks were ascribed tosignals from misoriented crystallites. (**d**) 2D-GIXD intensities of the spots corresponding to the denoted diffractions plotted as a function of the sample azimuthal angle. Cited from [37].

An intermolecular crystallographic structure of C_60_ on PnSC was analyzed in detail by GIXD. 2D-GIXD images of a PnSC sample covered with a 20 nm-thick C_60_ film deposited at room temperature are shown in Figure 4a–c. These images were acquired while rotating the in-plane azimuthal angle ϕ by 270° in 0.5° increments, and the image Figure 4a was obtained by integration of ϕ over 180°. Diffraction spots attributable to C_60_ $2\bar{2}0$ for its (111)-oriented fcc lattice appeared at ***q*** = (*q_xy_*, *q_z_*)~(12.5 nm^−1^, 0 nm^−1^), as shown in Figure 4b. These results indicated that C_60_ made physisorption on the PnSC surface to maintain its bulk crystal structure, and any chemical reactions such as photooxidation of C_60_ [61] and Diels–Alder adduct formation between C_60_ and pentacene [62] were minor, if any, for this heterojunction. When the sample was oriented to specific azimuthal directions, while those of PnSC 100 and $\bar{1}00$ were found at (*q_xy_*, *q_z_*)~(10.1 nm^−1^, 0 nm^−1^) in other directions (Figure 4c). Intensities of these spots and C_60_ $1\bar{1}1$ at (*q_xy_*, *q_z_*)~(7.2 nm^−1^, 2.5 nm^−1^) are plotted as a function of ϕ in Figure 4d. The PnSC 100 and $\bar{1}00$ spots blinked in a periodicity of 180° as expected from the symmetry of the crystal lattice. The six equivalent diffraction spots of C_60_ $2\bar{2}0$, that is, $2\bar{2}0$, $20\bar{2}$, $02\bar{2}$, $\bar{2}20$, $\bar{2}02$, and $0\bar{2}2$, appeared in periodicity of 60°, indicating the epitaxial growth of C_60_ on the PnSC surface. These ϕ-dependencies are visualized in a polar plot for *q_z_*~0 nm^−1^ (see Figure 2c).

**Figure 5:**
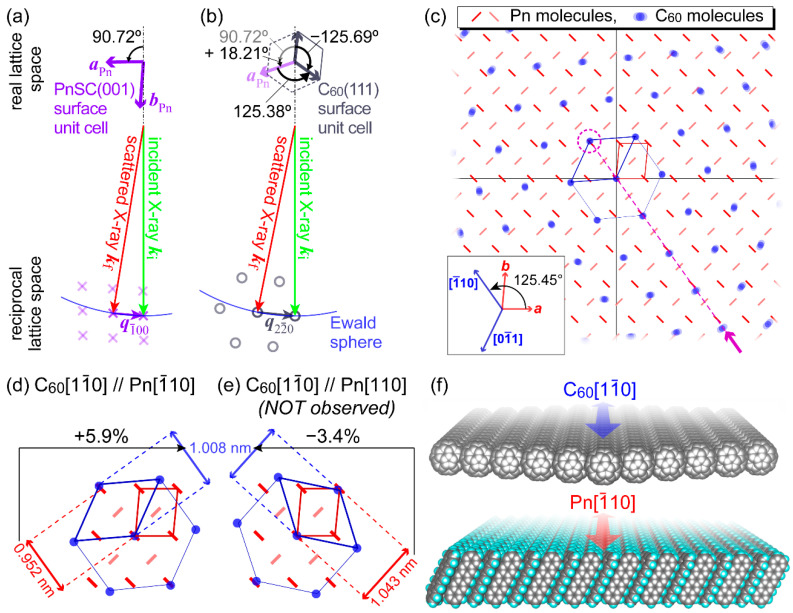
(**a**) In-plane orientation of the real space and reciprocal space lattices of the PnSC (001) surface at which the PnSC $\bar{1}00$ diffraction condition is fulfilled. Cross marks indicate the reciprocal lattice points of the PnSC (001) surface. (**b**) In-plane orientation of the real space and reciprocal space lattices of the C_60_ (111) surface at which the C_60_ $2\bar{2}0$ diffraction condition is fulfilled. The *a*-axis direction of PnSC at this orientation is also displayed for reference. Circles indicate the reciprocal lattice points of the PnSC (001) surface. (**c**) A schematic diagram representing an inter-lattice relationship between the PnSC (001) surface and the hetero-epitaxial C_60_ overlayer. (**d**) Lattice mismatch between the C_60_ (111) and PnSC (001) surfaces for the present inter-lattice orientation. (**e**) Lattice mismatch between the C_60_ (111) and PnSC (001) surfaces for a *hypothetical* inter-lattice orientation where C_60_ [$1\bar{1}0$ aligns with the [64] direction of the PnSC. (**f**) Schematic illustration of the molecular arrangements of the contacting layers at the C_60_/PnSC heterojunction.

By setting the sample orientation at ϕ where the PnSC $\bar{1}00$ and C_60_ $2\bar{2}0$ diffractions occurred, HR-GIXD measurements were conducted by using a NaI scintillation counter and Ge (111) analyzer crystal [63]. The intensity of the scattered X-ray was monitored during the fine rotation of ϕ to determine the exact orientations of the crystal lattices for both C_60_ and PnSC. The $\bar{1}00$ diffraction spot of the PnSC (001) surface appears in an in-plane sample orientation where the *a*-axis pointed 90.72° counterclockwise with respect to the X-ray incident direction as shown in Figure 5a. This azimuthal orientation is defined as ϕ = 0°. One of the C_60_ $2\bar{2}0$ spots appeared at which ϕ was rotated by (+18.21 ± 0.1)° from that where PnSC $\bar{1}00$ diffraction occurred. The orientation of the real and reciprocal lattices at ϕ = +18.21° is then illustrated as shown in Figure 5b. Taking the other five equivalent spots of C_60_ $2\bar{2}0$ into account, an inter-lattice relationship between the epitaxial C_60_ (111) and the (001) surface of PnSC was concluded as shown in Figure 5c. The adjacent C_60_ molecules align in a direction (125.45 ± 0.3)° counterclockwise to the *a*-axis of PnSC, which corresponds to the [$\bar{1}10$] direction of PnSC (125.606° counterclockwise to the *a*-axis). A lattice point of the C_60_ (111) surface roughly overlaps with the PnSC $\bar{1}10$ point with a lattice mismatch of less than 6% (Figure 5d). It is noteworthy that this inter-lattice orientation is not the best possible choice for the minimization of the lattice mismatch; in fact, the mismatch rate could be further reduced to about 3% if the nearest-neighbor C_60_ molecules aligned to the PnSC [110] direction instead (Figure 5e), any diffraction signals corresponding to such orientation of C_60_ crystallites were never detected. Another factor to be taken into consideration is the surface diffusion of ad-molecules on the surface, which takes place before the initial nucleation of the crystallites. Cantrell and Clancy predicted based on their molecular dynamics simulation results that the [$\bar{1}10$] direction is the most frequent axis for the C_60_ diffusion on the PnSC (001) surface [56]. This suggests a scenario in this epitaxial growth where the alignment of the molecular nuclei occurs along the molecular diffusion direction and this determines the orientation of the crystallites over the whole surface (Figure 5f).

**Figure 6:**
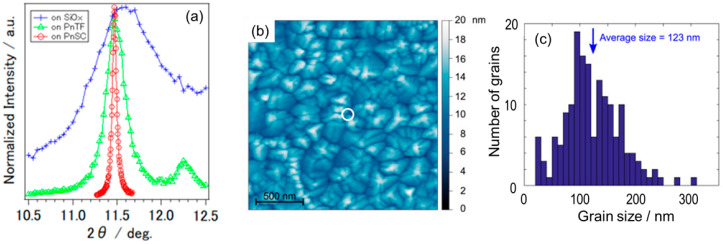
(**a**) 2θ profiles of the C_60_ diffraction spots formed on the single-crystal (red), polycrystalline thin film (green), and Si wafer (blue) substrates. (**b**) AFM image (2 µm × 2 µm) of a C_60_/PnSC sample. (**c**) Distribution of the grain size observed on the AFM image. The diameter of the circle at the center of the image illustrates the mean crystallite size estimated by HR-GIXD. (**b**,**c**) Cited from [65].

On the other hand, 2θ profiles of the C_60_ $2\bar{2}0$ diffraction spots were accurately measured by HR-GIXD to evaluate the in-plane mean crystallite size of the epitaxial C_60_ [65]. The full width at half maximum (FWHM) of the diffraction spot for C_60_ deposited on a PnSC substrate at 300 K was 0.0466° as shown in Figure 6a, from which the in-plane mean crystallite size of C_60_ was estimated to be (125 ± 7) nm. Since this size is in very good agreement with the average grain size (123 nm) of C_60_/PnSC obtained by AFM (Figure 6b,c), it can be inferred that each grain was composed of a single crystal domain of C_60_. It is noteworthy that the diffraction spots exhibited significant broadening for C_60_ deposited on polycrystalline pentacene thin films as well as on Si substrates directly (Figure 6a). This indicated that the usage of the highly ordered single-crystal pentacene, rather than disordered polycrystalline pentacene thin films, as a substrate, drastically enhance the crystallinity of the molecular heterojunctions.

**Figure 7:**
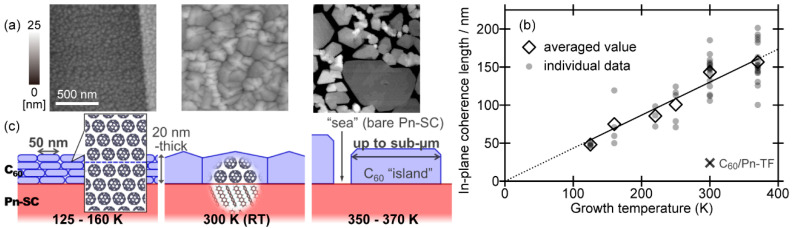
(**a**) AFM micrographs of 20 nm-thick C_60_ deposited on a PnSC substrate at various temperatures. (**b**) In-plane mean crystallite size (coherent length) of the 20 nm-thick C_60_ deposited on PnSCs plotted as a function of the growth temperature. The value for C_60_ on a pentacene thin film (PnTF) is also displayed for comparison. (**c**) Schematic drawings of growth manners of C_60_ on PnSC depending on the growth temperature. Cited from [66].

The C_60_ crystalline grain size can be further enhanced by an increase in the substrate temperature during the epitaxial growth [66]. Actually, AFM images (Figure 7a) revealed the emergence of widely extended islands with very flat tops and straight rims for samples grown at heated temperatures (370 K), whereas those for low temperature-grown samples (160 K) exhibited small particles dispersing over the surface. GIXD results confirmed that the epitaxial orientation of the C_60_ crystallites was independent of the growth temperature. The in-plane mean crystallite sizes derived from the C_60_ $2\bar{2}0$ spot profiles are plotted as a function of the growth temperature in Figure 7b, which disclosed almost proportional dependence of the in-plane C_60_ crystallite size to the growth temperature. Taking into account that the diffusion constant changes in the same dependence to the temperature under the Einstein–Smoluchowski relation, this fact also supports the aforementioned notion that the crystal growth of C_60_ on PnSC is dominated by the diffusion of adsorbed molecules on the substrate surface.

The growth manner of the C_60_ molecules on PnSC is summarized in Figure 7c. When the growth temperature was low (125–160 K), C_60_ formed relatively small crystallites. Out-of-plane XRD data on the low-temperature grown samples indicated that the mean crystallite size for the nominally 20 nm-thick C_60_ overlayers was about 5 nm, implying that the C_60_ crystallites were not coherent in the whole thickness range but included several discontinuities such as anti-phase domain boundaries. The increase in the growth temperature resulted in the extension of the C_60_ crystallites in both in-plane and out-of-plane directions, and even sub-micrometer-sized crystallites are available by moderate heating of the PnSC substrate during the epitaxial growth of C_60_.

It has to be mentioned that, although increasing the growth temperature is effective for the improvement of the grain size, this course soon faces a dead end because of the limited thermal stability of the PnSC substrate, such as the sublimation of pentacene molecules from the surface. In fact, a sudden drop of the in-plane mean crystallite size out of the proportional dependence to the growth temperature was observed upon further increase of the temperature above the data range plotted in Figure 7b [66]. Since low thermal stability is a common and innate property for organic semiconductors such as van der Waals molecular solids, epitaxial growth of further wider crystallite sizes even on molecular crystal substrates at room temperature or under moderate heating is highly anticipated. A potential route will be proposed later in Section 4.3.

### 3.2. Perfluoropentacene (PFP)/Pentacene Single Crystal

**Figure 8:**
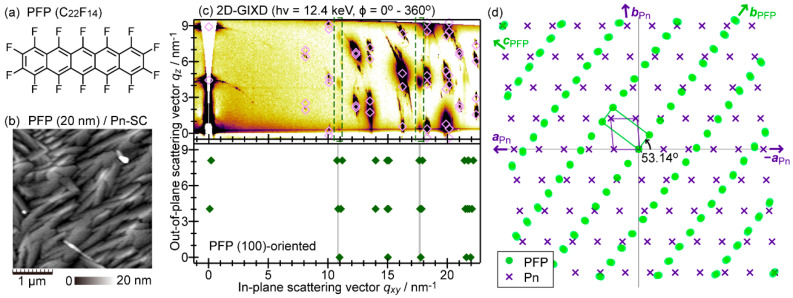
(**a**) Molecular structure of PFP. (**b**) An AFM micrograph of 20 nm-thick PFP on a PnSC sample. (**c**) Azimuthally integrated 2D-GIXD image of 20 nm-thick PFP on PnSC, and a simulated GIXD pattern for the PFP (100) surface. (**d**) Inter-lattice relationship between the epitaxial PFP and the PnSC ($00\bar{1}$) surface. Cited from [38].

Perfluoropentacene (C_22_F_14_, Figure 8a) is known as a “complementary” acceptor molecule sharing a common molecular skeleton with pentacene (C_22_H_14_) [67]. Contrasting to the case of the combination of pentacene and C_60_, pentacene and PFP tend to exhibit interdiffusion at their heterojunction due to the resemblance of their molecular structures [19,68,69]. However, very suggestively, it was unveiled that PFP crystallites align in the identical axis of the PnSC (001) surface to the aforementioned case of the epitaxial C_60_, despite the striking difference in molecular and crystallographic symmetries each other, as briefly introduced below.

The structures of PFP adlayers on PnSC substrates were prepared in the same manner as the case of C_60_/PnSC, and were determined by AFM and GIXD [38]. AFM images of a 20 nm-thick PFP-covered PnSC sample showed grains of quite an anisotropic shape that roughly pointed in a specific direction, as shown in Figure 8b, implying an occurrence of the epitaxial growth. Actually, 2D-GIXD data, indicating the formation of the (100)-oriented PFP crystallites with a known bulk crystal structure (*a* = 1.511 nm, *b* = 0.4490 nm, *c* = 1.1149 nm, α = γ = 90°, β = 91.567° [67]) as shown in Figure 8c, exhibited clear intensity variations of PFP-derived diffraction spots depending on the azimuthal angle of the sample. The inter-lattice relationship between the epitaxial PFP and PnSC was deduced as illustrated in Figure 8d. This means that the nearest-neighbor direction of the PFP molecules was aligned along with the [$\bar{1}10$] direction of PnSC, which was exactly the same as the case of the epitaxial C_60_ on PnSC.

An important characteristic of this epitaxial PFP/PnSC p–n junction is that the electronic band dispersion was demonstrated experimentally for both sides [70]. The energy-momentum dispersion of inter-molecular electronic bands was demonstrated to be 0.49 eV for the epitaxial PFP crystallites formed on PnSC. Notably, this bandwidth was as wide as that of single crystal rubrene (Section 4.1) which is well known as a high-mobility organic semiconductor material. The effective mass of holes at the valence band maximum was estimated to be similar to the electron rest mass *m*_0_. On the other hand, valence bands of PnSC had also been measured by ARPES to reveal a moderate hole effective mass (approx. 3.5 *m*_0_) [50,51]. These results suggest that the necessary conditions for band-like transport are fulfilled on both sides of this single-crystalline p–n heterojunction.

### 3.3. Tertaazanaphthacene (TANC)/Pentacene Single Crystal

**Figure 9:**
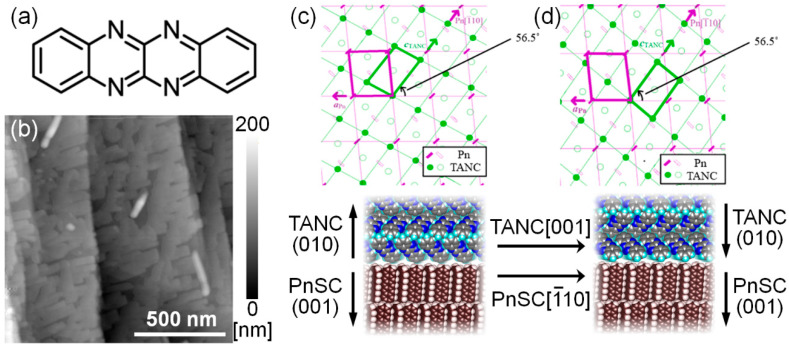
(**a**) Molecular structure of TANC. (**b**) An AFM micrograph of 20 nm-thick TANC on a PnSC sample. (**c**) Schematic drawings showing an inter-lattice relationship and cross-sectional molecular arrangements at the heteroepitaxial junction between the PnSC ($00\bar{1}$) surface and the “upward” TANC domain. (**d**) Inter-lattice relationship and cross-sectional molecular arrangements between the PnSC ($00\bar{1}$) surface and the “downward” TANC domain. The images of inter-lattice relations are cited from [78] (CC-BY).

Aza-substituted aromatic molecules have been of growing interest as a class of n-type small molecular semiconductors (acceptors) in recent years [71,72,73,74]. In this context, 5,6,11,12-tetraazanaphthacene (TANC, C_14_H_8_N_4_, Figure 9a) is one promising n-type material that is known to exhibit an n-channel operation as thin-film transistor devices [75] and its deep-lying HOMO-level [76]. While this material shares the same molecular symmetry as pentacene, the inter-molecular packing nature in its crystals is quite different from those in any known crystal phases of pentacene. Whereas herring-bone packing structures for pentacene are mainly dominated by so-called CH–π interactions, the leading factor for the TANC crystal structure is side-by-side CH-N hydrogen bonds which have been proposed as a key factor for designing high-performance n-type molecular materials [77]. TANC also exhibited the epitaxial growth on PnSCs in a similar but a little more complex manner to the case of PFP/PnSC [78].

AFM images of 20 nm-thick TANC on PnSC samples (Figure 9b) exhibited TANC islands of relatively uniform height (approx. 25 nm) and straight rims suggesting a formation of well-crystallized structures. An out-of-plane XRD profile on that sample clearly showed a peak at *q_z_*~8.45 nm^−1^, which corresponds to the 020-reflection of the known bulk crystal structure of TANC (*a* = 0.4710 nm, *b* = 1.491 nm, *c* = 0.7653 nm, α = γ = 90°, β = 94.701° [75]). In addition, under an assumption of the (010) surface of TANC, additional spots found in 2D-GIXD images were successfully assigned. These results indicated that TANC grew in the *b*-orientation of its bulk crystal structure on the PnSC surface. The 011/$01\bar{1}$ diffraction spots of TANC appeared only at specific sample azimuthal angles indicating the epitaxial growth of TANC. However, unlike the case of PFP/PnSC, the TANC 011/$01\bar{1}$ diffraction intensities exhibit four peaks (125.5°, 136°, 305.5°, and 316° from the angle where the $00\bar{1}$ diffraction of PnSC was detected) of two pairs with 180° separations. This means that the in-plane orientation of the TANC crystallites was not unique but two inequivalent crystalline domains facing different directions coexisted. The inter-lattice relationships between TANC and PnSC were derived from the 2D-GIXD results as shown in Figure 9c,d. In short, the two inequivalent domains of TANC corresponded to the “upward” and “downward” crystallites which mirrored against a common [001] axis of TANC aligning along the [$\bar{1}10$] direction of PnSC. It is noteworthy that the reference axis, i.e., PnSC [$\bar{1}10$], for the epitaxial growth was common in the cases of C_60_/PnSC and PFP/PnSC.

The electronic structures of the epitaxial TANC/PnSC heterojunction were also studied by photoelectron spectroscopy (PES). Unlike the case of PFP grown on PnSC, a sharp profile of the TANC HOMO-derived PES peak suggested a small energy dispersion width. The energy offset between the HOMO levels across this epitaxial heterojunction was derived to be 1.75 eV. The LUMO level of TANC was supposed to be just above the Fermi level, which corroborated the favorable character of TANC as good n-type material and the topical heterojunction can actually be used as a p–n junction of crystalline organic semiconductors.

## 4. Epitaxy in Various Types onto Rubrene Single Crystals

### 4.1. Heteroepitaxy of C_60_ Fullerene on Rubrene Single Crystal

In this subsection, we summarize reports on the crystal structures and qualities of interfaces of C_60_ on single-crystal rubrene as investigated by AFM, 2D-GIXD, and HR-GIXD.

**Figure 10:**
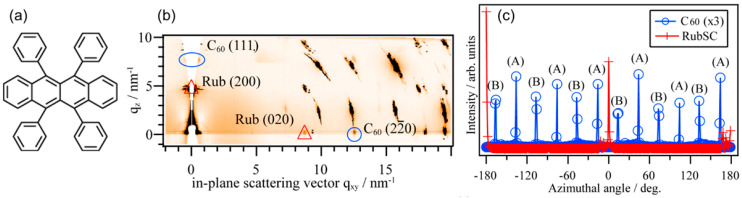
(**a**) Molecular structure of rubrene. (**b**) 2D-GIXD image of a C_60_/RubSC sample obtained by integration of the individual 2D-GIXD images over the sample azimuthal angle from 0° to 360°. Representative diffraction spots corresponding to the fcc-C_60_ and RubSC are marked with circles and triangles, respectively. (**c**) Diffraction intensities of C_60_ $2\bar{2}0$ and RubSC 020 plotted as a function of ϕ. The intensity for C_60_ is extended by a factor of three (**b**,**c**) cited from [40].

Rubrene (C_42_H_28_: Figure 10a) is a representative p-type organic semiconductor material with a very high hole mobility [79,80,81], and a long exciton diffusion length of 2–8 μm [82] in its single crystals. Rubrene is the first molecular species for which the inter-molecular valence band dispersion was experimentally observed in the bulk single-crystal samples [14,83], and accordingly, it has been regarded as a standard material for the scrutinization of the electronic structures in molecular crystals [84,85,86,87]. The crystal system is orthorhombic, with lattice constants of *a* = 2.69 nm, *b* = 0.718 nm, *c* = 1.44 nm [88], and the (100) plane with a rectangular unit cell appears on the surface [89]. In other words, this is a material with a higher symmetry than triclinic pentacene. Herein, we review how the epitaxial growth mode and crystallinity of the inter-molecular interfaces change when the symmetries of the crystal structure and molecular shape of the substrate organic single crystals are different.

The combination of rubrene and C_60_ has also been studied for a wide-variety of optoelectronic device applications [90,91,92,93,94,95,96]. Concerning the single-crystalline interfaces, Pinto et al. reported considerably high photoresponse at a heterojunction between [6,6]-phenyl-C_61_-butyric acid methyl ester (PCBM), a derivative of C_60_, and the single-crystal rubrene especially in the low energy region [97]. While they attributed the large photocurrent to enhanced exciton dissociation due to a polarization effect at the rubrene–PCBM molecular junction, detailed structures at the molecular contacts have not been disclosed experimentally. Fusella and coworkers proposed based on their results for C_60_ on polycrystalline rubrene thin films that photogeneration of the charge carriers can be enhanced by “band-like” delocalization of the excitonic states at the highly crystallized interfaces [98]. Although an AFM image presented in their article strongly implied the epitaxial growth of C_60_ on individual rubrene crystallites, detailed crystal structures of their heterojunction were hidden behind the polycrystalline nature of the rubrene substrates.

Mitsuta et al. reported the epitaxial growth of C_60_ crystallites aligning on the rubrene single-crystal (100) surface studied by reflection high-energy electron diffraction (RHEED), AFM, and GIWAXS [99]. They systematically measured AFM on samples with various deposition conditions (growth temperature and deposition rate) and concluded that higher substrate temperatures and slower deposition rates resulted in the C_60_ films of greater single-crystalline domains. The out-of-plane XRD profiles indicated that C_60_ grew in the (111)-orientation on RubSCs. RHEED patterns suggested the epitaxial growth of C_60_ on RubSC, and indeed GIWAXS results exhibiting the C_60_-derived diffraction spots at specific azimuthal orientations also confirmed this notion. However, dissimilar to the aforementioned C_60_/PnSC case, the C_60_ $2\bar{2}0$ diffraction peak was observed every 30°, indicating a kind of twelve-fold symmetry despite the six-fold symmetry of the C_60_ (111) surface. This means that C_60_ grew in two different growth orientations on the RubSC surface. These results were also found almost at the same time by GIXD works conducted by Tsuruta and coworkers [100]. Figure 10b,c shows an azimuthally integrated 2D-GIXD image and the in-plane orientation dependence of the C_60_ $2\bar{2}0$ and Rub 020 spot intensities for 20 nm-thick C_60_ thin films on a RubSC sample deposited at 300 K. The C_60_ $2\bar{2}0$-equivalent spots appeared in periodicity of approx. 30° were attributable to the presence of two-fold domains labeled as (A) and (B).

**Figure 11:**
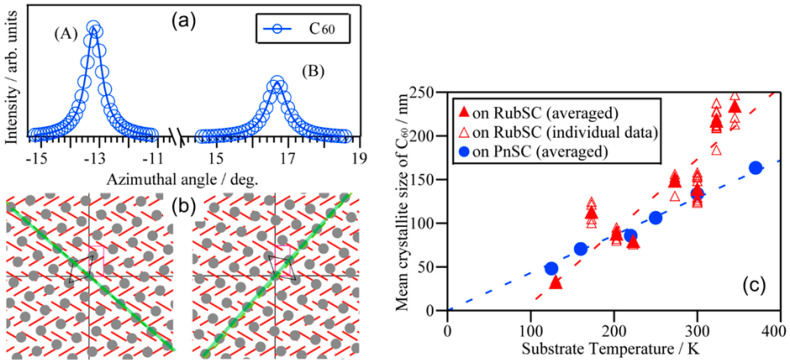
(**a**) Precise ϕ profiles of C_60_ $2\bar{2}0$ spots of the domains (A) and (B) taken by HR-GIXD. (**b**) Schematic diagrams representing inter-lattice relationships for the domain (A) and (B) of the epitaxial C_60_ on the RubSC (100) surface. The surface unit cells of C_60_ and RubSC are indicated by black- and red-colored lines, respectively, and the [$0\bar{2}1$] and [021] directions of RubSC for the (A) and (B) domains, respectively, are shown with green lines. (**c**) In-plane mean crystallite size of C_60_ on RubSC depending on the growth temperature derived from the HR-GIXD spot profiles of the C_60_ $2\bar{2}0$ spots. The temperature dependence for the C60 on PnSC case (same as the data shown in Figure 7b) is also displayed for reference. Cited from [40].

Orientations of these two types of crystalline domains were accurately determined through HR-GIXD measurements using a Ge (111) analyzer crystal [40]. The azimuthal orientation where the Rub 020 diffraction spot flashed was resolved at a fine ϕ-profile collected in 0.002° increments and was defined as ϕ = 0° hereafter. As shown in Figure 11a, ϕ-profiles of one of the C_60_ $2\bar{2}0$-equivalent spots measured in ϕ 0.1° increments gave peaks at ϕ = −13.23° and ϕ = +16.64° for the domains (A) and (B), respectively. The inter-lattice relationships for these two domains were derived as shown in Figure 11b. The C_60_ [$1\bar{1}0$] axis aligned along either RubSC [021] axis for the domain (B) or [$0\bar{2}1$] for the domain (A). The 2θ-profiles of the C_60_ $2\bar{2}0$-equivalent spots were identical for both domains. The in-plane mean crystallite size for C_60_ was evaluated to be about 125 nm independent of the domain orientation on the RubSC surface. This implies that adsorbed C_60_ molecules diffuse equally along crystallographically equivalent [021] and [$0\bar{2}1$] axes on the Rub (100) surface.

The in-plane mean crystallite size of C_60_ varied depending on the temperature of the RubSC substrates during the growth as shown in Figure 11c. Whereas the overall trend was similar to that of the C_60_/PnSC case, C_60_ on RubSC exhibited a steeper increase in the mean crystallite size as the growth temperature was elevated. Hence, the crystallite size of C_60_ was larger than that of on-PnSC in the high-temperature region above room temperature, and actually, highly crystalline domains with a mean size up to 250 nm were obtained on RubSC. On the other hand, in the low-temperature region, the in-plane crystallite size shrunk more rapidly and it may converge to zero at a finite temperature above 0 K. At present, the reasons for this discrepancy in the growth temperature dependence of the mean crystallite size between the C_60_/RubSC and C_60_/PnSC heterojunctions. One plausible factor to be considered should be packing densities at the surfaces. Ends of molecular backbones that are prone to congregate densely are directly exposed to the top surface of PnSC (001), whereas the tetracene backbones of rubrene molecules are buried beneath the phenyl side groups pointing to the surface. Actually, the density of benzene rings per unit surface area is about 6% smaller for RubSC (3.869 nm^−1^) in comparison to that for PnSC (4.123 nm^−1^). This variation in the surface molecular packing may affect the surface diffusion of the C_60_ molecules via the difference in vigor of molecular vibration at the substrate surfaces.

### 4.2. Homoepitaxy of Rubrene on Rubrene Single Crystal

Even though the aforementioned heteroepitaxial systems have demonstrated considerably good crystallinities at the molecular interfaces, some constraints in their crystal qualities are implied at the same time; e.g., judging from the growth temperature dependence represented as Figure 7b, a micrometer-scale mean crystallite size for C_60_ on PnSC is not realistic. One may recall orthodox doctrines for the epitaxial growth that minimization of a lattice-mismatch of an ad-grown material to a substrate crystal is a primary precondition for achieving a crystalline interface. Indeed, homoepitaxy, which in principle enables zero lattice-mismatch conditions between ad-layers and substrates, has succeeded in the fabrication of highly crystallized overlayers on metals and inorganic semiconductor materials [101,102,103]. To our knowledge, the first successful work for the homoepitaxy of organic molecular semiconductors was reported by Sassella et al. [104]. Rubrene was also known to grow homoepitaxially on bulk single-crystal and single-crystalline thin-film surfaces of rubrene [105,106], and as proposed by Hiramoto and coworkers afterward, it has opened a novel route for on-demand induction of charge carriers in bulk single crystals of high-mobility molecular semiconductors through chemical doping of impurities just like the manners for inorganic semiconductors [107,108,109]. Recently, Leo and coworkers have reported organic bipolar transistor devices consisting of homoepitaxial crystalline layers of rubrene grown on polycrystalline rubrene with a delicately tuned electrostatic potential distribution by sequential impurity doping [110].

**Figure 12:**
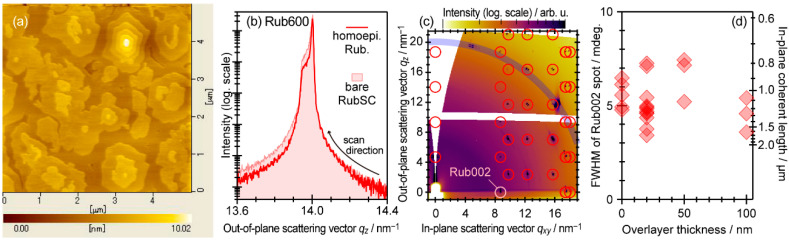
(**a**) AFM topography of a RubSC sample covered with 20 nm-thick homoepitaxial rubrene. Cited from [107]. (**b**) Out-of-plane diffraction spot profiles of a RubSC sample covered with 20 nm-thick homoepitaxial rubrene (red line) and a bare RubSC sample (light-red shaded). (**c**) Azimuthally integrated 2D-GIXD image of 100 nm-thick homoepitaxial rubrene on a RubSC sample. Simulated diffraction patterns for the RubSC (100) surface and Si (powder) are overlaid as red circles and a blue thick arc. (**d**) FWHM of the Rub002 diffraction spots plotted as a function of the homoepitaxial rubrene thickness. The right axis displays the corresponding coherent length for reference. The viewgraph (**d**) is cited from [31] (CC-BY).

Figure 12a shows an AFM image of 20 nm-thick ad-layers of rubrene formed on a RubSC substrate [107]. Micrometer-wide islands framed with monomolecular-height steps suggested the formation of highly crystallized overlayers. Actually, out-of-plane diffraction data (Figure 12b) indicated that no broadening of the Rub 600 reflection spot width for the homo-grown rubrene was confirmed in comparison to that of the bare RubSC (100) substrate; that is, the rubrene overlayers were coherently connected to the RubSC underneath. 2D-GIXD results also showed identical diffraction patterns to the bare RubSC up to the thickness of at least 100 nm (Figure 12c). These results clearly demonstrated that the homoepitaxial growth of rubrene on the RubSC surfaces actually occurred. In addition, for the Rub 002 diffraction spot widths, any signs of broadening were never detected irrespective of the rubrene overlayer thickness [31]. Whereas the observed in-plane spot width for the bare RubSC was mainly restricted by an angular resolution (~0.003° [63]) of the measurement system rather than the in-plane coherent length itself, the present results indicated that the in-plane mean crystallite size of the homoepitaxial rubrene was no smaller than the μm order.

AFM and GIXD results have confirmed that this excellent crystalline quality of the homoepitaxial rubrene was not substantially disturbed by doping of an inorganic acceptor material FeCl_3_ up to its ratio of 100 ppm [107]. The bulk doping at the doping rate of 10 ppm to the homoepitaxial rubrene achieved a concomitance of an undisturbed charge carrier mobility (several cm^2^V^−1^s^−1^) and significant dopant ionization rate (higher than 10%). Actually, the induction of holes at the valence band maximum was detected by high-sensitivity photoelectron yield spectroscopy (PYS) measurements. The combination of the accurate control of the ppm-level doping with the homoepitaxial growth of the single-crystalline organic semiconductors may lead to a new concept for an as-desired p/n switchover of high-mobility molecular semiconductors via the Fermi level tuning in the band gaps [64,109].

### 4.3. “Quasi-Homoepitaxy” of Di(Trifluoromethyl)Dimethylrubrene on Rubrene Single Crystal

In the previous subsection, it was introduced that homoepitaxial growth enables the formation of highly crystallized organic semiconductors with excellent coherent sizes of at least one order of magnitude greater than those of the heteroepitaxial molecular junctions. This concept can be adopted for the realization of single-crystalline organic semiconductor p–n homojunctions via bulk doping [108], just like in the case of Si. Nevertheless, it is still questionable whether such gradual p–n junctions can build sufficient electrostatic fields for driving efficient exchange between strongly bound molecular excitons and charge carriers for optoelectronic applications. For instance, orthodox organic photovoltaics generally demand abrupt energy level offsets at hetero-molecular junctions to overcome strong Coulombic attractions of electron-hole pairs by energetic gains accompanied by electron transfer from adjacent donor to acceptor materials.

**Figure 13:**
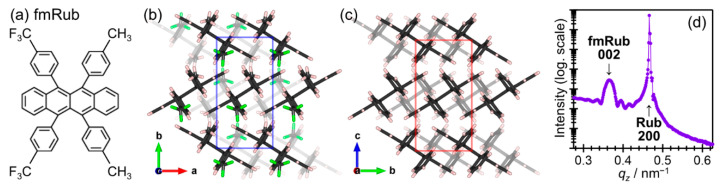
(**a**) Molecular structure of fmRub. (**b**) Molecular arrangement of the fmRub (001) surface. The surface unit cell is indicated as a blue rectangle. (**c**) Molecular arrangement of the RubSC (100) surface. The surface unit cell is indicated as a red rectangle. (**d**) Out-of-plane XRD profile of a RubSC sample covered with 50 nm-thick fmRub.

Are there any good tricks to satisfy both the excellent crystallinity of homoepitaxial organic semiconductor thin films and an abrupt electronic energy level offset at hetero-molecular contacts? Inorganic semiconductors have been developed by finding solutions for this problem: that is, looking for favorable combinations of target materials with crystalline substrates with minimized lattice mismatches. In this context, di(trifluoromethyl)dimethylrubrene (fmRub, Figure 13a), a derivative of rubrene, may be a promising molecule for a combination with (unsubstituted) rubrene because these two species have distinct frontier level energies with each other [111] but are sharing very close lattice constants in their “high-mobility” molecular planes (Figure 13b,c) [112]. In fact, fmRub formed a “quasi-homoepitaxial” interface on RubSC with an in-plane mean crystallite size several times greater than those for conventional heteroepitaxial molecular interfaces [39].

An out-of-plane XRD profile of a RubSC sample covered with fmRub by 50 nm showed a spiky peak attributed to the 200 reflection of RubSC and a broader one assignable to the fmRub 002 diffraction (Figure 13d). This indicated that the fmRub grew with its bulk crystal structure in the (001)-orientation onto the RubSC (100) surface. In addition, the fmRub-derived peak was accompanied by the Laue oscillation on both sides meaning uniformity of the crystallographic coherent length in the thickness direction.

**Figure 14:**
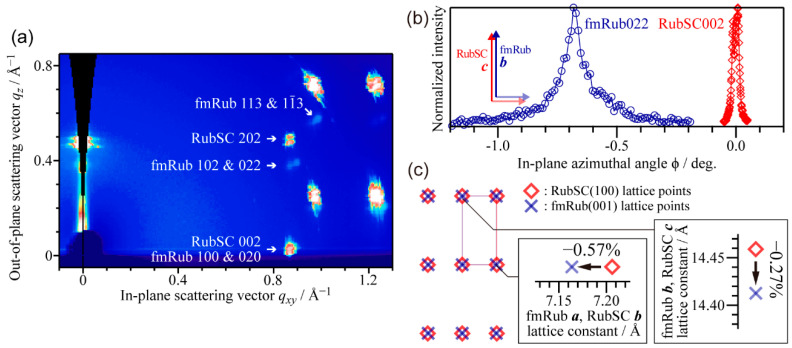
(**a**) Azimuthally integrated 2D-GIXD image of a RubSC sample covered with 50 nm-thick fmRub. (**b**) Azimuthal profiles of the RubSC002 (red) and fmRub022 (blue) diffraction spots. (**c**) Inter-lattice relationship between quasi-homoepitaxial fmRub and the RubSC (100) surface. Cited from [39] (CC-BY).

Figure 14a shows a 2D-GIXD image integrated over the sample azimuthal angle by 360°. Despite the close in-plane lattice constants of RubSC (100) and fmRub (001), one can distinguish diffraction spots of the latter from those of the former for non-zero *q_z_* ones of each. The fmRub-derived spots appeared only at specific azimuthal angles indicating the epitaxial growth of fmRub on RubSC. Actually, the azimuthal dependence of the GIXD profiles (Figure 14b) revealed that fmRub aligned its *b*-axis perfectly parallel to the *c*-axis of RubSC. Based on the lattice constants derived from the GIXD peak positions, an inter-lattice relationship between fmRub and RubSC was derived to be Figure 14c. These results demonstrated that the (001)-oriented fmRub grew “quasi-homoepitaxially” on the RubSC (100) surface.

It is noteworthy that this quasi-homoepitaxial molecular junction actually exhibited an improved crystallinity in comparison to the aforementioned heteroepitaxial cases. For instance, HR-GIXD 2θ scans revealed a fmRub 002 profile with a width of approx. 0.01° which corresponded to the mean crystallite size of 500 nm. This was considerably sharp in comparison to the standard heteroepitaxial ones: e.g., an average width of the $2\bar{2}0$-equivalent spots for C_60_ grown on RubSCs at RT was no narrower than 0.04° [40]. A micrometer-wide mean crystallite size, such as homoepitaxial rubrene, may be attained through further optimization of the growth conditions (e.g., growth rate and/or temperature) and refinement of the surface purity of the RubSC substrates. For the latter, it was reported that photo-oxidation of rubrene single-crystal surfaces forms bumps of a few nm in size, especially at the step edges [113], which may affect the molecular diffusion prior to the epitaxial growth. On the other hand, PES measurements confirmed the presence of a HOMO offset of 0.7–0.9 eV at this quasi-homoepitaxial junction. Moreover, upward energy shifts of the highest occupied states of both fmRub and RubSC as well as the vacuum level suggested an occurrence of band bending presumably caused by the formation of space charges via RubSC to fmRub charge transfer across the junction. After all, these results have certified that “structurally seamless but electronically abrupt” molecular junctions of organic semiconductors are actually enabled by quasi-homoepitaxial growth for the single-crystalline organic semiconductors.

## 5. Summary and Perspective

In this paper, recent works on epitaxial molecular junctions formed on two kinds of organic semiconductor single-crystal surfaces were reviewed. On the PnSC (001)/($00\bar{1}$) surfaces, three different molecular species, C_60_, PFP, and TANC, were revealed to grow in their own bulk crystal structures aligning each specific axis commonly to the PnSC [$\bar{1}10$] direction. On the other hand, three types of epitaxy, that is, heteroepitaxy, homoepitaxy, and quasi-homoepitaxy, were represented on the RubSC (100) surfaces by deposition of C_60_, rubrene itself, and fmRub molecules, respectively. In particular, it was proposed that the quasi-homoepitaxial hetero-molecular junctions may be promising for the concomitance of superior crystal qualities, which potentially fulfills a requirement for the band transport and abrupt electronic energy levels without any depletion regions for the organic semiconductor p–n junctions. These systems are prominent examples of the van der Waals epitaxy driven by weak inter-molecular couplings instead of covalent or ionic bonding, and the interface structures are formed on delicate balances between both weak admolecule–admolecule and admolecule–substrate interactions.

Toward applications of the well-ordered epitaxial molecular p–n junctions to emerging electronic devices such as organic lasers [114,115] and anti-ambipolar transistors [116,117,118] as well as OPVs [119,120], not only the structural knowledge and organization but also electronic and excitonic investigation and control are indispensable. As mentioned in each section, occupied electronic states for most of the epitaxial molecular junctions represented in this article were experimentally investigated. Nevertheless, it is still far from full understanding, and thus further multifold and integrated works are demanded. Although reliable photoelectronic measurements on bulk organic molecular single-crystal samples had long been unsuccessful subjects, the problems have been overcome by appropriate techniques: e.g., PYS for characterization of the highest-occupied electronic states of versatile insulator materials [121,122,123], highly sensitive photoelectron detection for the determination of electronic states at “buried” heterojunctions [124,125,126], and laser-assisted ARPES for mapping of the valence bands [14,123,127], which are ready for application (and in part have already been applied) to the epitaxial single-crystalline molecular junctions. In the next steps, the valence band mapping in the surface normal direction by excitation energy-dependence of ARPES [127,128,129,130], characterization of unoccupied electronic states by (angle-resolved) inverse photoelectron spectroscopy [131,132,133] or two-photon photoemission spectroscopy [86,134], and time/energetic properties of excitons by state-of-the-art electron measurement systems such as photoemission microscopy (PEEM) [134,135,136], two-photon photoemission techniques [134,137,138,139], high-sensitivity photoelectron detection [140,141,142,143,144], and angle-resolved measurements by electron energy-loss spectroscopy [145,146] are highly anticipated for pushing these epitaxial molecular junctions to the practical application stage.

On the other hand, one of many benefits of organic semiconductor applications to their inorganic counterparts is their processability through low-cost and resource-saving manufacturing from their solutions. Indeed, single-crystalline organic semiconductor thin films have been reported to form in macroscopic scales [147,148]. Although this review only focused on the molecular junctions produced totally in dry processes, i.e., gas-phase recrystallization of the single-crystal substrates and vacuum deposition of the ad-molecules, it does not mean that it is less important or infeasible to pursue solution-processable epitaxial p–n junction of molecular semiconductors. On the contrary, highly crystallized p–n junctions fabricated by wet processes have been of steady progress in these years [149,150,151,152,153]. Even for such more practical systems, further integration of the gas-phase epitaxy works, as the most idealistic cases, will give useful guidelines for smarter fabrication processes by providing molecular-scale insights into the growth manners of crystalline organic semiconductor contacts.

## Data Availability

The data presented in this study are available upon reasonable request from the authors.
